# Translation and Factor Analysis of the Stigma Scale for Chronic Illnesses 8-Item Version among Iranian Women with Breast Cancer

**DOI:** 10.31557/APJCP.2020.21.2.449

**Published:** 2020

**Authors:** Mona Daryaafzoon, Mohammadali Amini-Tehrani, Zahra Zohrevandi, Mehrnoush Hamzehlouiyan, Amene Ghotbi, Samira Zarrabi-Ajami, Hadi Zamanian

**Affiliations:** 1 *Faculty of Psychology, Islamic Azad University, Karaj Branch, Karaj, Alborz, *; 2 *Department of Psychology, Faculty of Psychology and Education, University of Tehran, *; 3 *Health Psychology and Behavior Medicine Research Group, Student Scientific Research Center, Tehran University of Medical Sciences, Tehran, *; 4 *Islamic Azad University, Roudehen Branch, Roudehen, *; 5 *School of Health, Qom University of Medical Sciences, Qom, Iran. *

**Keywords:** Breast cancer, psychometrics, stigma, translation, women

## Abstract

**Background:**

Cancer stigma is rarely addressed among Iranian population and patients. The current study aimed at translating and examining the construct validity of the stigma scale for chronic illnesses 8-item (SSCI-8) among Iranian women with breast cancer.

**Methods:**

In the current study, a total of 223 patients aged 19-75 years were recruited from three cancer centers in Tehran, Iran, from 2014 to 2015. Forward-backward translation method was used. The item-total correlation was evaluated. Exploratory factor analysis employing maximum likelihood method and direct Oblimin rotation was conducted. Reliability was assessed using composite reliability (CR). Average variance explained (AVE) was used for convergent/divergent validity.

**Results:**

The items mean was 1.47 (0.19), the scale mean 11.75 (5.57); the inter-item correlations were positive and significant (P <0.0001). A two-factor solution with seven eligible items (five for enacted and two for internalized stigma) showed the model fitness. The CR for the total scale, as well as enacted and internalized facets was 0.78, 0.89, and 0.79, respectively; the AVE was 0.66 for each latent variable.

**Conclusion:**

The Persian version of SSCI-7 was found as a reliable and valid abbreviated instrument to assess experiences of enacted and internalized stigma among Iranian women with breast cancer.

## Introduction

Breast cancer is the second leading cause of cancer death (Ferlay et al., 2015), with 2.4 million victims worldwide from 1990 to 2015 (Fitzmaurice et al., 2017). In Iran, most patients are in the age range of 40-50-year-old (Akbari et al., 2017), which is considerably lower than that of the developed countries (Nokiani et al., 2007; Valipour et al., 2017). Besides, the social context surrounding the patients may be contaminated with some negative attitudes against their illness (Boinon et al., 2014; Badihian et al., 2017). If the condition is revealed, such negative reactions, followed by the treatment of people, can be a source of fear or worry to patients with breast cancer (Nyblade et al., 2017). In spite of a relatively low negative attitude towards the blame for breast cancer in some contexts (Else-Quest et al., 2009), the provoked fear of discrimination and social isolation may harm the daily life of some other patients (Badihian et al., 2017). Importantly, the adverse effects and threat of the disease against the breast and body are of the concerns for the majority of the patients, which could bring about some internal distress resulting in negative body image perception (Kang et al., 2018) and intervening in their sexual life (Barthakur et al., 2017). Indeed, the feeling of embarrassment at the naked body may lead such patients to avoid sexual relationship and experience shame and distress (Mroczek et al., 2012; Gopie et al., 2013).

The concept of stigma as a social phenomenon explains the view of the society in which the pertinent condition is judged aversive, and the affected person is devalued in the eyes of others (Goffman, 1963; Noroozi et al., 2018). The stigma can threat the identity of the person, and it may be internalized in such a way that results in negative feelings and diminished self-esteem (Goffman, 1963; Corrigan and Watson, 2002). The cancer stigma, particularly, has drawn the attention to the detrimental burden of social views on the lives of some patients with cancer (Carter-Harris and Hall, 2014; Weiss et al., 2017; Bamidele et al., 2019; Dodd et al., 2019). The research so far consistently indicated that in spite of relatively low prevalence, the negative influence of any stigmatization is a part of sufferings imposed by the condition on the patients’ lives. Thus, any investigation about the effect of stigmatization and the rate of such experiences needs valid and reliable instruments.

Although some researchers highlighted the need of health care systems for advancement in provisions confronting the burden of stigma attached to the patients (Wilson and Luker, 2006), studies from Iran on patients with breast cancer rarely address stigma experiences. Studies on patients with chronic illnesses also entail a large number of questions and ideas; therefore, using short and brief instruments may help researchers to effectively spend the time and funds, and also consider the topics such as stigmatization, which needs more support from the part of the cancer care system, especially in Iran. 

The stigma scale for chronic illnesses 8-item version (SSCI-8) is a short-form instrument (Molina et al., 2013), which contains two facets of enacted and internalized stigmatization. Molina et al., (Molina et al., 2013) developed SSCI-8 in the USA for patients with neurological conditions (i.e., epilepsy, multiple sclerosis, Parkinson’s disease, stroke, and amyotrophic lateral sclerosis). The instrument is also used for patients with both neurological and physical conditions (Anagnostouli et al., 2016; Sarfo et al., 2017). This abbreviated instrument specifies common indicators of enacted stigmatization such as being shunned by others, feeling left out, and unkind or harsh treatments by others. The sense of embarrassment also as the internalized stigma is included to evaluate how the patients may feel about their physical limitations and illness.

Therefore, in response to the need for more evaluation of stigma among patients with breast cancer, which may help to find at-risk populations that need special care, the present study aimed at translating and evaluating the construct validity of SSCI-8 among Iranian women with breast cancer. This effort may provide a basis to help both care providers and researchers to address breast cancer stigmatization as an critical social issue in cancer care.

## Materials and Methods

*Design and sample*

In the current study, a total of 223 women were recruited from three cancer centers in Tehran, Iran, from December 2014 to February 2015. All the procedures in the current study were in accordance with the Declaration of Helsinki; in addition, the study protocol was approved by the Ethics Committee of. All participants provided the informed consent, could speak Farsi/Persian and received a breast cancer diagnosis at least one month preceding the study. The sample aged 19-75 years (mean = 47.10, SD = 9.10). Most women had “elementary to high school education” (n=181, 80.8%) and were married (n=181, 81.2%); the majority were a housewife (n=186, 83.4%) and urbanite (n=196, 87.9%). The household-level of income was mostly low (n= 116, 52.2%) followed by moderate (n=83, 37.3%). In average, more than 1.5 years (mean = 18.28, SD = 15.02 months) elapsed since the diagnosis of the sample. Most of the patients received chemotherapy (n=137, 61.4%) and underwent mastectomy (n=156, 69.9%).

*Instrument*

The SSCI-8 (Molina et al., 2013) evaluates stigmatization as a psychosocial concept referring to any act, thought, attitude, or perception toward a person with chronic conditions. It is derived from a 24-item questionnaire; SSCI-8 is an 8-item newly developed short-form instrument, which is appropriate for patients with chronic illnesses. The items are scored based on a five-point Likert scale from “never” to “always”; patients respond items based on their personal experiences, whether they subjected to any enacted stigma or felt stigma internally. The total score ranges from 8 to 40. Three questions (e g, “I felt embarrassed of my physical limitations”) are devoted to internalized stigma and other five ones to enacted stigma (e g, “People avoided me”). It was shown that SSCI short-form is a unidimensional scale, which its validity and reliability were satisfactory among patients of nine neurological illnesses (Molina et al., 2013).

*Translation procedure*

The forward-backward translation method was used for a valid translation of SSCI-8 into the Persian language (Sartorius and Kuyken, 1994). Two different translated versions of the instrument in Persian were provided by one of the first authors who had adequate and prior knowledge in the translation of English instruments, and a Ph.D. candidate in English language translation. The two initial drafts were checked by the principal researcher (corresponding author), which yielded the primary Persian draft of SSCI-8. The back-translation of the primary version was conducted by another Ph.D. candidate in English language translation, who had no previous familiarity with the scale. The back-translation showed accepted consistency of the primary draft with the original instrument in terms of conceptual and semantic equivalence (Sartorius and Kuyken, 1994). Five patients examined the face validity of the scale, and the results indicated its proper language and face validity. The procedure resulted in the Persian version of SSCI-8, included in the project’s questionnaire booklet. 

*Analysis approach*

Item statistics, including mean, standard deviation (SD), skewness, and kurtosis of the items, were reported. Item-total statistics were evaluated using inter-item correlations, item-total correlations, squared multiple correlations (SMC), and Cronbach’s alpha if any item deleted. The criteria including inter-item correlations and item-total correlations >0.2 as an indication of the least acceptable items’ interrelatedness for a broad construct and >.40 for a narrower construct (Clark and Watson, 1995; Morgado et al., 2017), squared multiple correlations (SMC) >0.4 as an indication of a fair amount of item’s variance explained by the other remaining items (equivalent to item’s communality) (Nunnally and Bernstein, 1994), and the conventional criterion of <0.1 increase in Cronbach’s alpha after deletion of any item were employed for evaluations.

To evaluate the construct validity of the scale, exploratory factor analysis (EFA) was performed employing maximum likelihood (ML) method of extraction followed by direct Oblimin rotation. The Chi-square test of goodness-of-fit with its degree of freedom and P-value was considered as a mean of model evaluation. The Kaiser-Meyer-Olkin test and Bartlett’s test of sphericity was conducted to ensure the legitimacy of factor analysis (Nunnally and Bernstein, 1994; Tabachnick and Fidell, 2013). In addition to eigenvalues above 1, the suggestion of scree plot among real dataset and the suggestion of parallel analysis (Watkins, 2005; Watkins, 2010) on random dataset also were adopted to find the eligible extracted factors. Reliability was tested using both conventional Cronbach’s alpha and composite reliability (CR) with the lowest threshold of 0.70. In addition, the index of average variance extracted (AVE) was used for further analysis of convergent/divergent validity of the scale (Hair et al., 2010; Malhotra and Dash, 2011).

**Figure 1:**
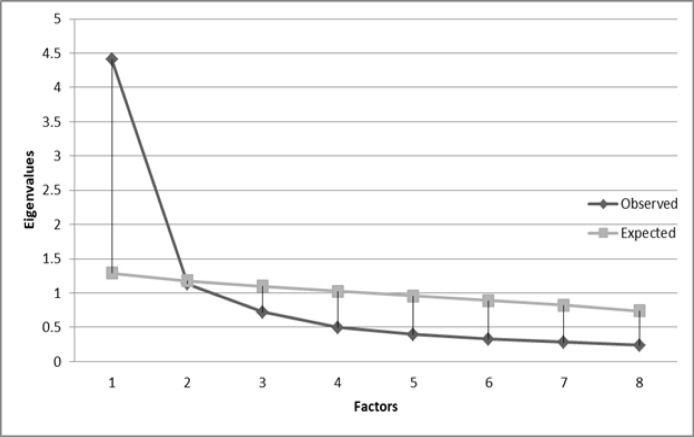
Results of EFA vs Parallel Analysis. Parallel Analysis was performed using Monte Carlo PCA for parallel analysis Software (Version 2.5.0.0; Watkins, 2010) with eight variables, 223 observations, and 1000 repetitions

**Table 1.** Item-Total Statistics of the Scale’s Items (n=223)

Statements	Mean (SD)	Skewness	Kurtosis	Corrected Item-Total Correlation	Squared Multiple Correlation	Cronbach's Alpha if Item Deleted
1-People avoided me^a^	1.37 (0.84)	2.4	5.36	0.635	0.586	0.85
2-I felt left out of things^b^	1.31 (0.81)	2.66	6.41	0.702	0.645	0.84
3-People avoided looking at me^a^	1.34 (0.84)	2.66	6.8	0.694	0.582	0.84
4-I felt embarrassed of my illness^c^	1.69 (1.17)	1.61	1.5	0.551	0.49	0.86
5-People seemed to be uncomfortable with me^a^	1.49 (0.94)	1.86	2.47	0.751	0.597	0.84
6-I felt embarrassed of my physical limitations^c^	1.76 (1.25)	1.51	0.98	0.596	0.526	0.86
7-People were unkind to me^a^	1.24 (0.70)	3.23	10.64	0.647	0.514	0.85
8-People acted as though my illness was my fault^a^	1.54 (1.07)	1.89	2.46	0.531	0.368	0.86
Total	1.47 (0.19)					0.87

(a), The statements originally regarded to enacted stigma; (b), The statement originally showed that belonged to both facets; (c), The statements originally regarded to internalized stigma (Molina et al., 2013).

**Table 2:** Exploratory Factor Analysis using Maximum Likelihood and Direct Oblimin Rotation (n=223)

Statements	EFA of 8 Items			EFA of Eight Items Forced to one-factor		EFA of Seven Items Forced to one-factor	
	Factor Loadings		Communalities	Factor Loadings	Communalities	Factor Loadings	Communalities
	Facet 1	Facet 2					
1-People avoided me^a^	0.878	-0.119	0.668	0.776	0.601	0.788	0.621
2-I felt left out of things^b^	0.871	-0.035	0.726	0.827	0.684	0.839	0.703
3-People avoided looking at me^a^	0.783	0.03	0.64	0.802	0.644	0.806	0.65
4-I felt embarrassed of my illness^c^	0.025	0.737	0.565	0.502	0.252	0.493	0.243
5-People seemed to be uncomfortable with me^a^	0.65	0.225	0.636	0.802	0.643	0.791	0.626
6-I felt embarrassed of my physical limitations^c^	-0.028	0.886	0.758	0.533	0.284	0.514	0.264
7-People were unkind to me^a^	0.705	0.019	0.513	0.719	0.517	0.71	0.504
8-People acted as though my illness was my fault^a^	0.337	0.299	0.315	0.54	0.292		

(a), The statements originally regarded to enacted stigma; (b), The statement originally showed that belonged to both facets; (c), The statements originally regarded to internalized stigma. EFAs were performed using maximum likelihood method of extraction with Oblimin rotation. Factor loadings were reported as Pattern Matrix suggested. Communalities are reported as extraction. Facet 1 refers to extracted enacted stigma, and Facet 2 refers to extracted internalized stigma (Molina et al., 2013).

**Figure 2:**
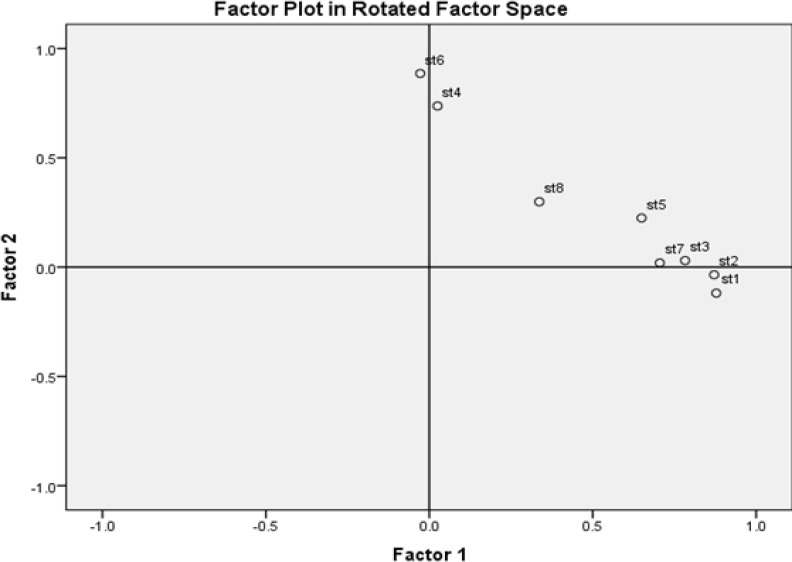
Factor Plot which Indicates that Item 8, Regarded to the Fault of the Illness, was not a Part of Any Facets of Stigmatization among Breast Cancer Patients

## Results

*Item-total statistics*

Table 1 represents the statistics and item-total properties of SSCI-8 scale. The items mean (SD) was 1.47 (0.187), and the scale mean (SD) was 11.75 (5.57). The inter-item correlations were significant (P <0.0001) ranging from 0.29 to 0.73 with a mean (SD) of 0.480 (0.134). This indicated that all items were positively inter-related from low to relatively high intensities. Also, the item-total correlations ranged from 0.53 to .075, all of which exceeded the desirable criteria for the constructs (Clark and Watson, 1995; Morgado et al., 2017). In addition, the SMCs indicated that item #8 (SMC = 0.37; ‘People acted as though my illness was my fault’) regressed on all remaining items was accounted by the relatively least amount of variance explained (Nunnally and Bernstein, 1994). Furthermore, the Cronbach’s alpha for eight items was 0.89, which would be dropped slightly if any item deleted. The skewness ranged 1.51 (item #6: ‘I felt embarrassed of my physical limitations’) to 3.23 (item #7: People were unkind to me) and the kurtosis 0.98 (item #6) to 10.64 (item #7).

*Exploratory factor analysis*

The KMO was 0.852, and Bartlett’s test of sphericity was almost significant; Chi-square (28) = 908.645, and P <0.0001. These results legitimated the conduction of EFA on the sample. Parallel analysis with eight variables, 223 observations, and 1,000 repetitions suggested that four factors with eigenvalues of 1.289, 1.1788, 1.099, and 1.025 could be extracted in a random dataset. The EFA with ML followed by direct Oblimin rotation indicated two factors with respective eigenvalues of 4.450 and 1.133, which could contribute to 55.07% and 14.16% of the variance observed, respectively (total variance observed = 69.23). The two factors were correlated with the intensity of 0.56, P <0.01. However, according to Figure 1, the second extracted factor could hardly compete with the results of parallel analysis, based on the fact that its eigenvalue was less than an estimated eigenvalue in a random dataset. Figure 1 shows the corresponding eigenvalues of both EFA and Parallel Analysis. 

Table 2 presents the results of EFA. In terms of factor loadings, items #1, 2, 3, 5, and 7 were loaded strongly on the first factor, all of which address enacted stigma. The second factor consisted of items #4 and 6 regarding the embarrassment of illness and physical limitations, respectively; both of which address the internalized stigma. The result indicated that item #2 that was correlated with both facets of stigmatization in the study of Molina et al., (2013), was clearly loaded along with items of the enacted stigma. In addition, item #8 regarding ‘the fault of the illness,’ was not loaded higher than the accepted threshold for robust scales (i e, >0.4) (Tabachnick and Fidell, 2013). According to Table 2, the initial EFA showed that communality of all legitimate items could contribute significantly to the variance of the construct, except item #8 with a communality of 0.315. Likewise, the results of the structure matrix indicated that this item has a similar correlation with both factors (0.50 and 0.49, respectively). It is more evident in factor plot shown in Figure 2. The goodness-of-fit for this solution indicated that the model was not properly fit to the data (Chi-square (13) = 42.172, P-value <0.0001).

To test whether the deletion of item #8 could provide better fitness, EFA was conducted on the remained seven items. This resulted in a well-fitted model with Chi-square (8) =13.666, P-value = 0.091. The Cronbach’s alpha of these two factors was 0.89 and 0.79, respectively showing adequate internal consistency. All seven items (with the deletion of item #8) had an internal consistency of 0.86.

To test the unidimensionality of the SSCI-8 and -7, EFA was forced to extract one factor. According to Table 2, all items could be loaded from fair to strong on the factor, although the communality of the items of internalized stigma (items #4 and #6) as well as item #8 were low (0.252, 0.284, and 0.292, respectively). These results indicated fairly low contributions to the variance of the one-factor construct. Altogether, these eight items could explain 48.965 of the total variance in a single factor. Besides, this model was not fit to data; Chi-square (2) = 147.658, P-value <0.0001.

Another EFA was forced to extract one factor, including seven eligible items; again, similar results indicated the inadequacy of a one-factor solution. Here, the factor loading of both items #4 and 6 was relatively low and their contribution to the total variance was weak (with communality of 0.243 and 0.264, respectively). This model did not also fit to the data; Chi-square (14) = 113.619, P-value <0.0001. However, these seven items could better explain the total variance with 51.59% on a one-factor structure than the eight-item structure.

*Reliability*

According to Hair et al., (2010), reliability in terms of internal consistency can be better examined using the CR index with a lower threshold of 0.7, in addition to the AVE index with a lower threshold of 0.5 for a robust scale. Thus, AMOS software (v. 24, IBM Inc.) was employed to model the one-factor stigmatization (including seven indicators), and two covariated facets of enacted stigma (including five indicators) and internalized stigma (including two indicators). The CR for the total scale, as well as the enacted and internalized facets was 0.78, 0.89, and 0.79 respectively, indicating sound internal consistency. The AVE for the total scale, as well as the enacted and internalized facets (r = 0.57, P-value <0.0001), was respectively 0.66, 0.66, and 0.66, indicating that a valid amount (>50%) of each latent variable’s variance was explained by their corresponding observed variables (i.e., items). Therefore, according to the results, the Persian version of SSCI-7 had desirable psychometric properties among Iranian women.

## Discussion

The present study aimed at translating and examining the construct validity of SSCI-8 among Iranian patients with breast cancer. The study population included Iranian patients with mostly low to moderate educational level recruited across a wide age range of 19 to 75 years. In addition, their social context was relatively less educated with only a few mothers/husbands with higher education. A majority of them also underwent partial or complete mastectomy.

The results of EFA revealed that the scale was bi-dimensional with a clear distinction between the enacted and internalized items. In contrary, the original study by Molina et al., using item response theory indicated unidimensionality of the scale among patients with different neurological disorders (Molina et al., 2013). A study from Korea aiming to validate the scale among patients with neurological disorders also yielded a unidimensional factor structure encompassing both enacted and internalized stigma (Yoo et al., 2017). This disparity suggests that the stigmatism of different conditions might entail dissimilar structures, as it contains differences in terms of impact (Else-Quest et al., 2009). It also might suggest that there is a distinction between the way “people” express their negative attitude towards a given condition and the affected individuals, and the reaction by which the patients show how they feel about their diminished self (i e, self-stigma). This distinction might be justified due to a causal relationship in which enacted stigma from social context may bring about low self-esteem (Corrigan and Watson, 2002), which may be expressed through feelings of shame and embarrassment (Parrott et al., 1988; Velotti et al., 2017).

One another inconsistency of the study results with those of the stud by Molina et al., (2013) was on item #2, “Because of my illness, I felt left out of things”. In their work, Molina et al., (2013) found that this item is loaded on both enacted and internalized stigma components. In contrast, the current study findings demonstrated that this particular item clearly addresses an enacted stigmatization, at least in the context of breast cancer. This finding means that item #2 might refer to the social distancing experienced by Iranian women with breast cancer.

Also, the item #8, “People acted as though my illness was my fault,” indicated a weak contribution to the scale variance with a fairly poor factor loading. In other words, Iranian women with breast cancer did not associate blame for their condition with stigmatization in any sort. Also, item-total statistics, specially SMCs, suggested that the item #8 either had a relatively lower inter-relatedness to the underlying construct (Nunnally and Bernstein, 1994) or it could be determined by other relevant experiences not captured in the scale, including self-blame as an indication of self-stigmatization. Nevertheless, this finding might suggest that the attribution of blame for breast cancer to the patients might not be a significant experience related to stigmatization, as suggested by a study comparing the role of self-blame among patients with breast, lung, and prostate cancers (Else-Quest et al., 2009). These results led the authors to eliminate this item from further analysis of the validity and reliability of the Persian version of SSCI-7. However, it is recommended evaluating item #8 in other populations with different contextual backgrounds. 

In terms of reliability, the study results indicated that SSCI-7 has desirable internal consistency both in total scale and the subscales. For the total scale, it was consistent with the results of the original study (Molina et al., 2013) and the study on Korean patients with neurological disorders (Yoo et al., 2017). Moreover, the AVE index above 0.5 for the total scale and each subscale demonstrated that each item could contribute to a high amount of variances of the scale (Hair et al., 2010). Especially for the internalized stigma subscale, this acceptable AVE indicated that the subscale with only two items would be reliable in further research due to its relatively high communality (Worthington and Whittaker, 2006).

However, the study also had some limitations. The results necessarily need further analysis for cross-validation (Thompson, 2004). The study sample was not representative of the whole population of Iranian women with breast cancer, which limits the generalizability of the findings. Also, the study sample had mainly elementary to high school level of education; thus, they may represent the population with lower levels of literateness, which could affect the readability of the questionnaire and the subsequent results. However, because face-to-face method (interviewer-administered) was utilized for completing the questionnaires, It may argue that the possible bias due to the sample’s comprehension deficiencies was reduced. Nevertheless, cautions should be considered in interpreting the results and generalizing the findings to populations with higher levels of education. Importantly, one might argue that there was a lack of addressing to some contextual aspects of stigmatization in Iranians daily living; a crucial issue. Besides, the self-report nature of the study represents the perceived evaluation of the respondents of how they were treated as a result of their condition (i.e., breast cancer). This suggests that their perceptions might be a biased report of the actual acts of stigmatization. Thus, caution is needed in translating the results into the rate of social stigmatization upon the patients with breast cancer. 

The Persian version of SSCI-7 with seven items can be used as a reliable and valid abbreviated instrument to assess the experience of enacted and internalized stigma among Iranian women with breast cancer. Authors suggest employing the full scale if it is used in an aggregated fashion; albeit, a confirmatory factor analysis as a cross-validation technique is needed to evaluate the performance of the scale with either eight or seven items. Since research on psychosocial issues of patients with cancer is mainly focused on the significant challenges with high-pitched expression, incorporating this abbreviated instrument with desirable psychometric properties and adequate content in the projects can enable the researchers to address the most prominent, but covert, aspects of the cancer experience, the stigma.
